# Systematic Review of Scales for Measuring Infectious Disease–Related Stigma

**DOI:** 10.3201/eid3003.230934

**Published:** 2024-03

**Authors:** Amy Paterson, Ashleigh Cheyne, Benjamin Jones, Stefan Schilling, Louise Sigfrid, Jeni Stolow, Lina Moses, Piero Olliaro, Amanda Rojek

**Affiliations:** University of Oxford, Oxford, UK (A. Paterson, A. Cheyne, B. Jones, S. Schilling, L. Sigfrid, P. Olliaro, A. Rojek);; Tulane University, New Orleans, Louisiana, USA (J. Stolow, L. Moses);; Global Outbreak Alert and Response Network, World Health Organization, Geneva, Switzerland (J. Stolow, L. Moses);; The Royal Melbourne Hospital, Melbourne, Victoria, Australia (A. Rojek)

**Keywords:** COVID-19, Ebola virus, HIV/AIDS and other retroviruses, communicable diseases, disease outbreaks, epidemics, prejudice, social discrimination, social marginalization, social stigma

## Abstract

Infectious disease outbreaks are associated with substantial stigma, which can have negative effects on affected persons and communities and on outbreak control. Thus, measuring stigma in a standardized and validated manner early in an outbreak is critical to disease control. We reviewed existing scales used to assess stigma during outbreaks. Our findings show that many different scales have been developed, but few have been used more than once, have been adequately validated, or have been tested in different disease and geographic contexts. We found that scales were usually developed too slowly to be informative early during an outbreak and were published a median of 2 years after the first case of an outbreak. A rigorously developed, transferable stigma scale is needed to assess and direct responses to stigma during infectious disease outbreaks.

Infectious disease outbreaks are typically accompanied by stigma (*1*–*4*). Stigma can be defined as the denial of social acceptance to a person or group due to an attribute deemed discrediting by their community or society (*5*,*6*). That umbrella term includes the cognitive or affective endorsement of negative stereotypes, referred to as prejudice; negative behavioral manifestations, referred to as discrimination; and medically unwarranted avoidance or neglect of affected persons (*6*,*7*) (Figure 1).

**Figure 1:**
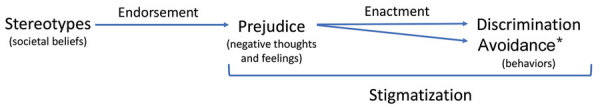
Conceptualization of stigma used in a systematic review of scales for measuring infectious disease–related stigma. Graphic is based on N. Jones and P.W. Corrigan (*6*) and M.G. Weiss (*7*). Asterisk (*) indicates cases where avoidance is medically unwarranted.

Stigma associated with infectious disease outbreaks reduces affected persons’ opportunities for physical, social, and psychological well-being, contributing to social and health inequalities (*8*–*11*). COVID-19 and Ebola virus disease (EVD) stigmatization have specifically been proven predictors of severe psychological distress, depression, anxiety, and posttraumatic stress disorder symptoms (*1*,*11*–*13*). Stigma can also impede efforts to control disease outbreaks by fueling fear, decreasing uptake of preventive measures (including vaccination), discouraging health-seeking behavior such as seeking testing and treatment, and reducing adherence to care (*6*,*8*,*10*,*14*).

Furthermore, outbreak-related public health interventions can affect the stigma associated with a disease (*10*). In a systematic review of the psychological effects of quarantine, persistent stigma was a central theme (*15*). Contact tracing has been found to lead to linear blaming of affected persons (*10*). Vaccination status can be a source of social stigma (*16*–*18*), as can decisions about mask-wearing (*19*). Although evidence of the exacerbation of stigma might not fully undermine the value of these public health interventions, those outcomes highlight the need for the inadvertent social consequences to be considered and minimized where possible.

A range of stigma reduction interventions have been described in the literature (*6*–*8*,*14*). However, without robust stigma scales, determining where these interventions are most needed and evaluating their effectiveness in outbreak settings is difficult (*11*). Stigma scales have been used in other infectious disease contexts (most routinely HIV) and could be similarly helpful when applied to emerging and re-emerging disease outbreaks (*11*). 

We identified disease-associated stigma scales used in outbreak settings and described the commonalities, strengths, and limitations of those scales. The results of this review are intended to improve the development and use of stigma scales in infectious disease outbreaks and inform the design of a transferable scale that can be used across different infectious disease outbreaks.

## Methods

### Review Strategy 

We conducted a review to determine what scales have been used for measuring stigma due to outbreaks in affected communities through January 31, 2023. We assessed the common content themes within those scales; methods used to develop and validate scales; psychometric properties (i.e., validity and reliability) of available scales; transferability of scales; and limitations in the development, validation, and use of those scales. 

We defined an outbreak as a rapid, unexpected increase in disease case numbers. Therefore, stigma associated with endemic, chronic diseases, such as HIV and tuberculosis, were outside the scope of this review. 

We reported this review in line with the PRISMA (Preferred Reporting Items for Systematic Reviews and Meta-Analyses) 2020 checklist (*20*). Our review was informed by the COSMIN guideline for systematic reviews of patient reported outcome measures (*21*). The review protocol is registered on PROSPERO (registration no. CRD42023396387).

### Search Strategy and Eligibility Criteria

We formulated a search strategy with a librarian. The search strategy combined terms for the key components “stigma,” “infectious disease outbreaks,” and “prevalence scale” by using the Boolean operator “AND” (Appendix Figure). We searched MEDLINE, PsycINFO (https://www.apa.org/pubs/databases/psycinfo), CABI Global Health, Embase, Web of Science, and Cochrane Library databases with no language restrictions. We retrieved all records published though January 31, 2023. We also screened bibliographies of relevant systematic reviews and included additional studies that met the eligibility criteria.

### Study Selection

We assessed the retrieved records according to our eligibility criteria (Table 1). We uploaded all citations to EndNote 20.5 (https://endnote.com) and removed duplicates, after which we uploaded titles and abstracts to Rayyan systematic review software (https://www.rayyan.ai). Two independent reviewers screened a random 10% of titles and abstracts and we used Cohen’s kappa (κ) to calculate inter-rater reliability. For conflicts, the 2 reviewers discussed the studies and agreed or asked a third reviewer to provide a final decision, then clarified or refined the eligibility criteria. We repeated this process until κ showed excellent agreement (*22*), after which all further titles and abstracts were divided and screened by 1 reviewer.

**Table 1:** Eligibility criteria used in a systematic review of scales for measuring infectious disease–related stigma

Criteria	Inclusion	Exclusion
Population	Involved community members of any age affected by infectious disease outbreaks with or without a personal history of the disease	Focused exclusively on healthcare workers
Concept	Described the development, validation, or use of a stigma scale, such as a survey, questionnaire or other instrument consisting of >2 closed-end questions that form a composite score and aim to measure outbreak-related stigma prevalence	Focused on broader measurements of intersectional stigma during, but not due to, the outbreak of concern*
Context	Related to infectious disease outbreaks	Focused on non-communicable diseases or chronic infectious diseases
Study types	Cross-sectional or cohort studies	Interventional studies without a pre-intervention survey
	Studies describing scale development, piloting, or validation	Studies investigating stigma exclusively through qualitative methods
	Interventional studies which include pre-interventional surveys providing observational data.	Protocols, guidelines, book sections, case-reports, opinion pieces (editorials, viewpoints, commentaries) conference abstracts, preprints, and unpublished literature
Minimum validity of scale	Use of stigma scales that, at a minimum, have been assessed for face validity†	Not applicable

*Includes scales that assessed stigma associated with race, sexual orientation, mental health, weight, or class during an outbreak or epidemic but not in direct relation to the outbreak disease. For example, scales that assessed race-based discrimination unrelated to association with COVID-19 during the pandemic. †For instance, scales were at least superficially reviewed by potential end-users, experts, or both to confirm that the scale appears to reflect the concept of stigma in the relevant contexts (*21*).

The reviewer screened eligible full text publications by using the same process. We achieved the required κ after the second round of title and abstract screening (κ = 0.76) and the second round of full text screening (κ = 0.82). Where complete stigma scales were not available, we emailed corresponding authors to request access. If the scale was still not provided, we excluded the study. For non-English stigma scales, we used a professional translation service to translate the scale into English (Appendix). Where multiple articles described the same study activities, we included the article with the most available information on the relevant stigma scale.

### Data Extraction and Analysis

One reviewer extracted data by using Excel 2021 (Microsoft, https://www.microsoft.com). Another reviewer independently extracted a random 10% sample of the data to ensure reliability.

We assessed the psychometric properties (i.e., validity and reliability) of scales according to COSMIN guidelines (*21*) (Table 2). We assessed transferability for each scale by using a previously described cross-cultural equivalence framework (*23*) (Appendix Table 1).

**Table 2:** Definitions of psychometric properties used in a systematic review of scales for measuring infectious disease–related stigma*

Domain	Property	Aspect of property	Definition
Validity			The degree to which an instrument measures the constructs it purports to measure
	Content validity		The degree to which the content of an instrument is an adequate reflection of the construct to be measured
		Face validity	The degree to which an instrument looks as though it reflects the construct to be measured
	Construct validity		The degree to which the scores of an instrument are consistent with hypotheses (for instance regarding internal relationships, relationships to scores of other instruments, or differences between relevant groups) based on the assumption that the instrument validly measures the construct to be measured
		Structural validity	The degree to which the scores of an instrument are an adequate reflection of the dimensionality of the construct to be measured
		Hypotheses testing	The degree to which the scores of an instrument are consistent with hypotheses on relationships to scores of other instruments
		Cross-cultural validity	The degree to which an instrument accurately measures the same construct in different population groups.
	Criterion validity†		The degree to which the scores of an instrument are an adequate reflection of a gold standard*
Reliability			The degree to which the measurement is free from measurement error
	Internal consistency		The degree of the interrelatedness among the items
	Test-retest reliability		The amount of the total variance in two sets of measurements which is due to 'true’ differences between respondents
	Measurement error		The systematic and random error of a respondent's score that is not attributed to true changes in the construct to be measured
Responsiveness			The ability of an instrument to detect change over time in the construct to be measured

*Table adapted from COSMIN definitions of domains, measurement properties, and aspects of measurement properties, which uses the term “gold standard” (*21*). †Criterion validity assessment was not considered in this review because no standard for stigma assessment is available.

We used framework synthesis to identify the domains of stigma included in the scales (*24*). That method of evidence synthesis is used increasingly for health-related reviews and combines framework and thematic analysis techniques (*24*). The method involves starting with an a priori conceptual framework and coding all included studies against that framework (*24*). New themes, or in this case stigma domains, are generated from evidence not captured by the a priori framework (*24*). The approach thereby adopts a mixed deductive and inductive approach to produce a revised conceptual framework (*24*).

We used a previously developed stigma typology (*6*) as the a priori framework for our analysis (Appendix Table 3). We then adjusted and added to the framework throughout the analysis as new domains emerged that were not captured by the existing framework. For example, many scales included questions about stigmatization by employers and coworkers but did not fit into the existing framework; therefore, we added a new domain, termed workplace stigma, to the framework. All authors discussed and agreed upon each addition or adjustment to the framework. We used the same approach for identifying themes in acknowledged limitations.

### Quality Assessment

We assessed the quality of each study by using the COSMIN Risk of Bias Checklist (*25*). That checklist uses a modular approach dependent on whether the study was intended for scale development or validation and the aspects of the scale the study set out to validate. The quality of each relevant method is given a rating using by using a worst score counts principle (*25*).

## Results

**Figure 2:**
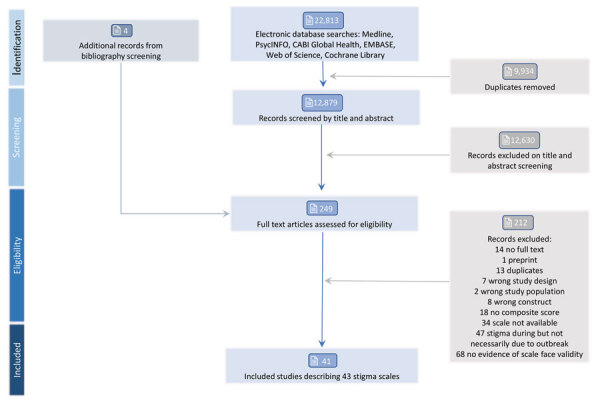
Diagram of studies included in and excluded from a systematic review of scales for measuring infectious disease–related stigma. Reviews were performed in accordance with PRISMA (Preferred Reporting Items for Systematic reviews and Meta-Analyses) guidelines (*20*). PsycINFO is a database of the American Psychiatric Association (https://www.apa.org/pubs/databases/psycinfo).

Our search strategy retrieved 12,879 records after deduplication (Figure 2). We excluded most records at title and abstract screening because the search term “discriminat*” referred to the discriminatory ability of prediction models or tests, rather than social discrimination. 

We found 249 records eligible for full-text review. Of those, we found 41 studies that described the development, validation, or use of 43 unique outbreak disease­–associated stigma scales that met the inclusion criteria. We included those 43 scales in this review.

### Overview of Scales

Of the 43 included scales, 42 (98%) were newly developed specifically for the outbreaks of concern (Appendix Table 4); 38 (88%) were used only once in the published literature. The scales were used in 27 different countries.

Thirty-two (74%) scales focused on COVID-19–associated stigma, 7 (16%) assessed EVD-associated stigma, 2 (5%) were SARS-associated, and 1 (2%) scale each was used in Lassa fever, long COVID, and Zika virus disease. Those scales were published a median of 25 (interquartile range 18–30) months after the first case of a given outbreak.

Almost half (21 [49%]) of the scales were based on HIV literature and existing HIV stigma scales (Appendix Table 4). Only 9 (21%) scales included primary qualitative data in the scale development processes. The Long COVID Stigma Scale (*26*), was the only scale explicitly codeveloped with affected community members.

### Content of Scales

**Figure 3:**
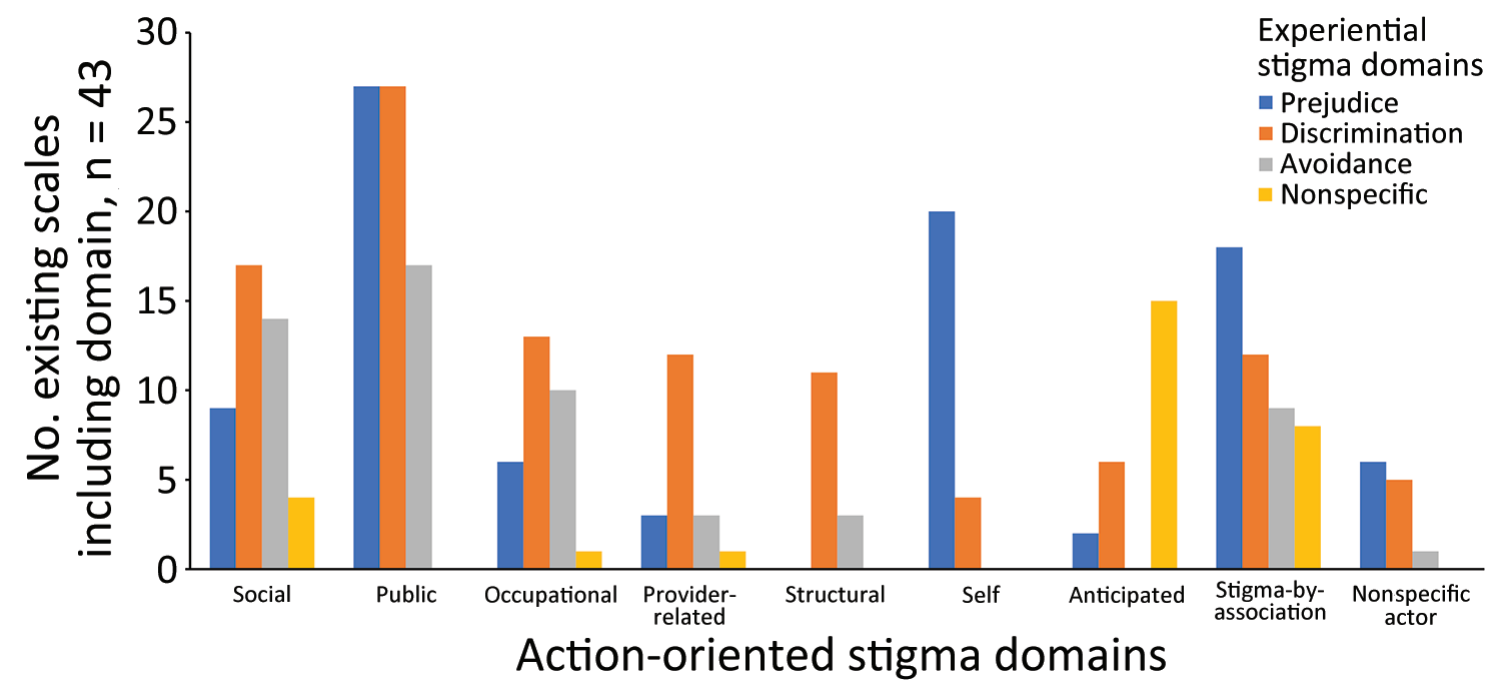
Frequency of inclusion of domains of stigma in a systematic review of scales for measuring infectious disease–related stigma. Graph displays existing scales from framework synthesis. Action-oriented stigma domains included the following: social, stigmatization by friends and family; public, stigmatization by broader community and strangers; occupational, stigmatization by colleagues and employers; provider-related, stigmatization by service providers; structural, stigmatization by institutions; self, internalized stigma; anticipated, disclosure concerns or avoidance due to fear of stigma; nonspecific actor, item does not specify who is enacting stigma.

We identified 24 domains of stigma in the included scales by using the framework synthesis process (Table 3). Those domains included 3 distinct stigma experiences: prejudice, discrimination, and avoidance of persons beyond suggested public health measures. Those stigma experiences were enacted by different groups, including family and friends (social stigma), broader community and strangers (public stigma), colleagues and employers (occupational stigma), service providers (provider-related stigma), and institutions (structural stigma). Our final framework also included the internalization of stigma (self-stigma), avoidance of stigma (anticipated stigma), and stigmatization of persons associated with the disease but not directly infected (stigma-by-association). The most common domains were public prejudice, public discrimination, and self-prejudice. Provider-related, occupational, and anticipated prejudice were infrequently included in the scales (Figure 3).

**Table 3:** Definitions and example scale items for each domain identified in a systematic review of scales for measuring infectious disease–related stigma*

Action-oriented stigma domains†	Experiential stigma domains		
	Prejudice‡	Discrimination§	Medically unwarranted avoidance¶
Social: stigmatization by friends and family	“I feel blamed by relatives or friends,” Self-stigma Scale (SSS-15)	“[I was] forced to change residence because of social alienation,” 7-item EVD-related stigma index	“People I cared for stopped calling or interacting after learning that I was infected/suspected,” COVID-19 Stigma Scale
Public: stigmatization by broader community and strangers	“Most people think that a person who has had Ebola is disgusting,” Ebola/COVID-19–related Stigma Survey	“I have been insulted/discriminated because of my history of being infected/suspected,” COVID-19 Stigma Scale	“Some people avoid touching me even after my recovery once they knew I was infected with/suspected,” COVID-19 stigma scale
Workplace: stigmatization by colleagues and employers	“My feeling of job security has been affected by my illness,” COVID-19 Perceived Stigma Scale-22 (CPSS-22)	“I will dismiss my employee who recovers from COVID-19,” Social stigma and discriminatory attitudes scale	“Someone refused to buy products from you,” Stigmatization related to EVD and COVID-19 scale
Provider-related: stigmatization by service providers	“You feel it is not worthwhile for you to serve persons who contracted COVID-19” - Stigma Discrimination Scale (SDS-11)	“[I was] treated unfairly by healthcare professionals,” COVID-19 Experienced DISCrimination Scale (CEDISC)	“I was denied health care services when the doctors found out I was infected /suspected,” COVID-19 Stigma Scale
Structural: stigmatization by institutions	NA	“The first COVID-19 patient in each city should be identified and penalised due to their role in spreading the disease,” COVID-19-related enacted Stigma Questionnaire	“At the hospital/clinic, I was made to wait until the last,” Ebola-related stigma instrument
Self: internalization of stigma	“Having had COVID-19 infection makes me feel that I am a bad person,” COVID-19-related Stigma Survey	“I stopped eating with other people,” Ebola-related stigma instrument	NA
Anticipated; disclosure concerns or avoidance due to fear of stigma	“I worry that people may judge me negatively when they find out I have long Covid,” Long COVID Stigma Scale (LCSS)	“You have avoidance behaviours such as staying home for fear of being stigmatised or rejected,” Stigmatization related to EVD and COVID-19 scale	NA
Stigma-by-association; stigmatization of those societally associated with the disease or infected persons but not personally infected	“If they knew about it would your neighbors, colleagues or others in your community think less of your family because of your COVID-19 infection?” Arabic Explanatory Model Interview Catalogue (EMIC)	“A school refused to accept your children,” Stigmatization related to EVD and COVID-19 scale	“If a person was infected with COVID-19, it is better to avoid his/her family members,” Community COVID-19 Stigma Scale

*Framework based on stigma typology from Jones and Corrigan (*6*). EVD, Ebola virus disease; NA, not applicable. †Domains adopted from Pescosolido and Martin (*27*). ‡Negative thoughts and feelings toward stigmatized persons. §Enactment of prejudice or differential treatment of stigmatized persons. ¶Neglect of stigmatized persons.

More than one quarter (14 [28%]) of scales included items that deviated from widely accepted definitions of stigma, including the definition used in this review (Figure 1). Those scales considered adoption of recommended preventive measures (e.g., people should stay away from those infected with COVID-19) and limited knowledge of disease (e.g., COVID-19 only affects the elderly) as evidence of stigmatization.

Sixteen (37%) scales asked participants whether they endorsed or participated in stigmatization toward others, 15 (35%) ask about participants’ own experiences of stigmatization, and 4 (9%) enquired about participants’ observations of stigmatization toward others in their community. Eight (19%) scales included items from a mixture of those perspectives.

### Psychometric Evaluation of Scales

Psychometric evaluation (i.e., assessment of validity and reliability) of scales was notably limited (Appendix Table 5). Among the scales that underwent validation processes, none consistently met the COSMIN criteria for sufficient validity and reliability (*21*).

Approximately half (24 [56%]) the scales were assessed by both relevant professionals and community members before administration. Only 3 studies (*28**–**30*) reported formal content validity scores. According to the COSMIN criteria (*21*), all scales had indeterminate or inconsistent content validity by our definitions (Table 2).

Among included scales, 20 (47%) had been tested for structural validity, and 12 (60%) met the COSMIN criteria for sufficient validity (*21*). Five (12%) scales had been evaluated for construct validity using hypotheses testing, all of which met the sufficiency criteria (*21*). Six (14%) scales had been assessed for test-retest reliability, and 3 (50%) were deemed sufficient (*21*). No studies assessed responsiveness, that is, the ability of an instrument to detect change in a construct over time (*21*).

For 32 (74%) scales, authors had reported on internal consistency, and most used Cronbach α coefficients. However, because the structural validity of a scale needs to be confirmed before internal consistency can be tested (*21*), we could only consider 17 (53%) of those scores. Of those 17 scales, 4 (24%) had α<0.7, suggesting inadequate internal consistency (*31*).

### Transferability of Scales

Only 1 scale, the Stigmatization Related to EVD and COVID-19 Scale (*1*), was used across different outbreaks. However, that scale is not publicly available, and we had to request it. In addition, the COVID-19–Related Stigma Survey administered in India and Bangladesh (*32*,*33*) is closely related to the Ebola-Related Stigma Scale administered in Liberia (*34*) and adopted 14 of the original scale’s 16 items. Three scales were administered in >1 country. Six scales were used across different participant profiles (i.e., community members with and without lived experience of the disease). No scales had sufficient evidence of cross-cultural equivalence when we reviewed them using a cross-cultural equivalence framework (*23*) (Table 4).

**Table 4:** Transferability of scales determined by a systematic review of scales for measuring infectious disease–related stigma

Scale name	Transferability		
	Cross-national	Cross-outbreak	Participant profile†
Stigmatization related to EVD and COVID-19 scale	Used; IE	Used; IE	Not used; A
Ebola-related Stigma Scale	Not used; U	Not used; A	Not used; A
COVID-19­–related Stigma Survey	Used; IE	Not used; A	Not used; A
COVID-19 Stigma Scale	Not used; U	Not used; U	Not used; A
Community COVID-19 Stigma Scale	Not used; U	Not used; U	Not used; A
7-item EVD-related Stigma Index	Used; IE	Not used; A	Used; IE
Eight-item Stigma Scale	Not used; U	Not used; A	Not used; A
Arabic Explanatory Model Interview Catalogue (EMIC)	Not used; U	Not used; U	Not used; A
COVID-19 Stigma Instrument-Patients (CSI-P2)	Not used; A	Not used; A	Not used; A
The Perceived Courtesy Stigma Sub-scale	Not used; U	Not used; A	Not used; U
The Affiliate Stigma Sub-scale	Not used; A	Not used; U	Not used; A
Modified 12-item HIV Stigma Scale	Not used; U	Not used; A	Not used; A
Ebola-related Stigma Instrument	Not used; A	Not used; U	Not used; A
Stigma Discrimination Scale (SDS-11)	Not used; U	Not used; A	Used; IE
Self-stigma Scale (SSS-15)	Not used; A	Not used; A	Not used; A
COVID-19 Bullying Scale	Not used; U	Not used; U	Used; IE
COVID-19 Experienced DISCrimination Scale (CEDISC)	Not used; U	Not used; U	Not used; A
Covid-19 Internalised Stigma Scale (COINS)	Not used; U	Not used; U	Not used; A
COVID-19 Responsibility Attribution Scale	Not used; A	Not used; A	Not used; A
COVID-19 Attitudes Scale	Not used; A	Not used; A	Not used; A
SARS Social Life and Services Stigma Self-report Questionnaire	Not used; A	Not used; A	Used; IE
SARS Discrimination in the Workplace Self-report Questionnaire	Not used; A	Not used; A	Used; IE
Stigma toward EVD Survivors Scale	Not used; U	Not used; U	Not used; U
EVD Stigma Index	Not used; U	Not used; U	Not used; A
COVID-19-related Enacted Stigma Questionnaire	Not used; A	Not used; A	Not used; A
Discrimination in Medical Settings Scale	Not used; U	Not used; U	Not used; A
30-item Bullying during the COVID-19 Pandemic Questionnaire	Not used; A	Not used; U	Not used; U
Stigmatising Attitudes Scale	Not used; A	Not used; A	Not used; A
COVID-19 Stigma Scale (COVID19SS)	Not used; A	Not used; U	Not used; U
COVID-19 Perceived Stigma Scale-22 (CPSS-22)	Not used; U	Not used; U	Not used; A
Public Attitudes toward Stigma Questionnaire	Not used; A	Not used; A	Not used; A
Perceived Stigmatization of COVID-19 Scale	Not used; A	Not used; A	Not used; A
Modified Version of the KAP Survey Tool on Zika Virus Disease	Not used; U	Not used; U	Not used; U
Public COVID-19-related Stigma toward Patients Measure	Not used; U	Not used; U	Not used; U
Public COVID-19-related Stigma toward Wuhan People Measure	Not used; A	Not used; A	Not used; U
EVD-related Stigma Scale	Not used; A	Not used; U	Used; IE
COVID-19 Public Stigma Scale	Not used; U	Not used; A	Not used; A
Social Stigma and Discriminatory Attitudes Scale	Not used; U	Not used; U	Not used; A
Long COVID Stigma Scale (LCSS)	Not used; U	Not used; A	Not used; A
Modified Measure of Disease-Related Stigma (MDRS) Scale	Not used; A	Not used; A	Not used; A
Lassa Fever-associated Stigmatization Scale	Not used; U	Not used; A	Not used; U
The Social Stigma Scale	Not used; A	Not used; A	Not used; A
COVID-19 related Social Stigma Scale	Not used; A	Not used; A	Not used; A

*Insufficient evidence (IE) indicates insufficient evidence of cross-cultural equivalence and transferability as assessed using cross-cultural equivalence framework devised by S.A.M. Stevelink and W.H. Van Brakel (*23*) (Appendix Table 1, https://wwwnc.cdc.gov/EID/article/30/3/23-0934-App1.pdf). A, substantial adaptations anticipated for cross-cultural use; U, appears readily usable. †Usability for persons with and without a personal history of the disease.

### Acknowledged Limitations of Included Studies

Authors of the included studies commonly acknowledged inadequate validation of the stigma scales as a limitation. Most studies also noted the inability to establish causality because of the adoption of a cross-sectional study design. In addition, more than half of the studies expressed concern about the generalizability of their findings because they used nonrepresentative sampling techniques and had undercoverage bias for certain subpopulations.

### Quality Assessment of Studies

For 35 studies that described scale development, we found that 7 (20%) received a doubtful quality rating for those methods according to the COSMIN Risk of Bias Checklist (*25*), and we rated the rest inadequate (Appendix Table 5). We found similar ratings for studies that aimed to content validate an existing scale. Conversely, we found that structural validity, internal consistency, test-retest reliability, and hypotheses testing methods more commonly received very good or adequate quality ratings, but those methods were infrequently conducted.

## Discussion

We found that numerous scales have been developed to assess outbreak-related stigma and that those scales have been used in a wide range of geographic settings. That finding illustrates a global recognition and concern about the stigma associated with infectious disease outbreaks and potential adverse impacts of stigma. However, shortcomings in the development, validation, and use of those scales mean that stigma is being incompletely and unreliably measured during outbreaks and that comparison of experience across outbreaks is not possible.

We found that, according to the COSMIN Risk of Bias Checklist (*25*), the quality of scale development and content validation methods were inadequate or doubtful for all studies. Similarly, several other forms of psychometric assessment (e.g., test-retest reliability) were not performed on most scales, which could be because of shortcuts taken in best practices in research methods because of the perceived urgency of an outbreak. However, those shortcuts compromise the validity of study findings. Thus, psychometric validation using best-practice guidelines (*31*,*35*) should be more rigorously applied to stigma scales and routinely reported. Of the scales reviewed, the Perceived Courtesy Stigma Scale and the Affiliate Stigma Scale (*36*) had the most evidence of sufficient validity and reliability, although the content and cross-cultural validity and responsiveness should be assessed during future use of those scales.

In addition, we noted a lack of repeated use of scales across diseases and settings, despite similarity in scale content and derivation from the same HIV-related stigma scales. That finding represents a missed opportunity to maximize scale development efforts, strengthen the evidence base of a scale, and expand understanding of the common impacts of stigma across outbreaks (*11*,*14*,*18*).

The fact that half the scales were derived from HIV scales also raises concerns about scale validity when applied to acute outbreaks. For example, stigma-by-association questions specific to sexual partners or groups at high risk for HIV infection might not be appropriate in other outbreaks. Similarly, questions about avoidance might not account for mandated isolation of affected persons in certain outbreaks, which could explain the misuse of items such as “people should stay away from those infected with COVID-19” and other key preventive measures as markers of stigma in more than one fourth of scales we reviewed. That misuse could be avoided by adopting theoretical frameworks in scale design by using formal content validity scoring processes (*31*) and ensuring that the scales are informed by qualitative data from in-depth or semistructured interviews with end users and other stakeholders (*25*).

Stigma scales tended to capture more advanced forms of stigmatization, such as public discrimination and the internalization of persistent stigma (i.e., self-stigma). Poor detection of the potential precursors of those forms of stigma, such as social, occupational, or provider-based prejudice, were not investigated; however, if identified, those precursors could be targeted before action, thereby reducing the detrimental effects of stigma on outbreak control and patient well-being (*8*).

In addition, the high frequency of stigma-by-association as a theme in the reviewed scales recognizes that noninfected community members are not only potential stigmatizers but might also be stigmatized. Therefore, the current practice, which gives scales about stigma experiences to persons who have had the disease but gives noninfected community members scales asking about endorsement of stigma, is a false dichotomy. Persons can be both a stigmatizer and be stigmatized (*8*). That false dichotomy could be overcome by using items that are distanced (i.e., less personal) from the respondent, such as case vignettes or questions about third-person observations (*37*). Those types of items enable all community members, regardless of disease status, to answer a wider range of questions while reducing social desirability bias. Another option, drawing from the HPTN 071 (PopART) trial (*38*), is to use multiple scales in parallel to separately ask persons with lived experience of the disease, healthcare workers, and other community members about experienced and endorsed stigma.

Of note, the median time from the start of an outbreak to publication of a relevant stigma scale was 2 years. That timeframe can be partially attributed to the traditionally slow peer-reviewed publication process, which is a recognized obstacle to efficient translational science in emerging outbreaks (*39*). However, the delay can also be attributed to the lengthy process involved in stigma scale development and implementation, which often results in outbreak-related stigma being investigated retrospectively, rather than early in an outbreak, when the scale has the greatest potential to inform response interventions and risk communication. The lack of early identification of stigma is also a major omission in the existing research because evidence suggests stigma can be most detrimental early in an outbreak because of heightened isolation (*3*,*10*).

Together, our findings demonstrate that the model of de novo scale development for each outbreak does not work in the context of emerging infectious diseases and leads to small, overlapping, methodologically weak, and slow outcomes, despite the best intentions of developers. As is the case with clinical research on emerging diseases (*39*), overcoming the challenge of stigma scale development requires an innovative approach.

A critical need exists for preemptive development of a methodologically rigorous stigma scale that can be easily adapted for new outbreaks. Such a scale would enable outbreak responders to immediately integrate stigma assessment into surveillance activities at the onset of an outbreak. That measure should be developed or endorsed by international and national public health institutions to ensure adequate funding and reach of the scale, aid in cross-learning, and reduce duplication of efforts.

The feasibility of a standardized scale is supported by the similarities in stigma manifestations across disease and geographic contexts. Those similarities are noted both in this review and in previous stigma literature (*8*,*11*,*14*). A modular approach to the scale, whereby additional context- and disease-specific items can be included as appropriate, could capture stigma specific to distinct outbreak settings.

Within pandemic preparedness in other fields, such as vaccine development and clinical research, efforts to ensure rapid outbreak response includes solving for disease X, a hypothetical, undefined pathogen of potential consequence (*40*). We suggest the preemptive stigma scale development and validation process mirror that process.

To optimize adoption and usefulness, a stigma scale needs to be publicly available and used in longitudinal, preinterventional, and postinterventional studies, rather than restricted to cross-sectional use. In turn, results of those studies need to be effectively disseminated to policymakers, response actors, and affected communities, which could inform the adaptation of response interventions to minimize associated stigma (*8*,*10*).

The limitations of this systematic review include that the screening strategy relied on inclusion of stigma or a similar term in the title or abstract. Therefore, studies that used a stigma scale but did not report it in their abstract might have been missed. Second, because the review was not limited to scales in the English language, the local meaning and relevance of some of the items might have been distorted with translation. Finally, this review did not include healthcare worker­–specific scales, which might more frequently include occupational- and provider-related stigma items. Nonetheless, this review included an extensive search of the literature, without language or date restrictions, and provides a meaningful summary of the uses, validity, and transferability of existing outbreak stigma scales. 

In conclusion, rapid and methodologically sound assessment of stigma is a critical and urgently needed aspect of outbreak response. This review demonstrates a range of readily implementable improvements that could be made to outbreak stigma scale design and use (Table 5). The data and recommendations we provide can be used to design valid and versatile stigma scales for ongoing and future outbreaks.

**Table 5:** Recommendations for future outbreak stigma scales determined by a systematic review of scales for measuring infectious disease–related stigma

Area	Recommendations
Design	A theoretical framework of stigma should be applied from conception of the scale to ensure all relevant domains of stigma are represented.
	Future scales should be co-designed with persons with lived experience of outbreak-associated stigma.
	Scale items should be informed by qualitative research alongside existing scales.
	When resources allow, scale design should be informed by a range of outbreak diseases and settings to enhance transferability of the scale. This should be facilitated by large public health institutions.
	Established best practices for ensuring cross-cultural equivalence (e.g., [*23*]) should be followed when translating and adapting scales for cross-contextual use.
Validation	Scale items should be formally assessed for content validity (including clarity, relevance, and comprehensiveness) by both experts in the field and relevant community members with lived experience of stigma.
	Confirmation of the structural validity of scales should precede internal consistency testing. Other forms of reliability, including test-retest reliability, should be routinely assessed alongside internal consistency.
	The cross-cultural validity of scales should be assessed across countries, diseases, and respondent profiles using multi-group factor analyses or Differential Item Functioning analyses.
	The responsiveness of scales should be assessed to ensure they have the ability to detect changes in stigma over time.
Use	Scales should be used in longitudinal and pre- and post-interventional studies to assess stigma trends over the course of an outbreak, rather than limited to cross-sectional use.
	When possible, representative sampling techniques should be adopted in administration of stigma scales.
	The results of studies assessing stigma during outbreaks, as well as the stigma scales used, need to be rapidly publicly disseminated with minimal access barriers such as paywalls.

Appendix Additional information on a systematic review of scales for measuring infectious disease–related stigma.
