# Supplementary material for: Systematic Review of Scales for Measuring Infectious Disease–Related Stigma
Source: Emerg Infect Dis. 2024 Mar;30(3):519–29. doi: 10.3201/eid3003.230934 (PMC10902531; doi:10.3201/eid3003.230934)
Supplement: Appendix — Additional information on a systematic review of scales for measuring infectious disease–related stigma. [file 23-0934-Techapp-s1.pdf]

Article DOI: https://doi.org/10.3201/eid3003.230934

EID cannot ensure accessibility for supplementary materials supplied by authors. Readers who have difficulty accessing supplementary content should contact the authors for assistance.

Systematic Review of Scales for Measuring Infectious Disease–Related Stigma

Appendix

Translation Process

Only one scale did not have an English version provided online or by the authors on request. This was the COVID-19 related Social Stigma Scale which was provided in Arabic. Gengo, a paid professional translation company, was used to translate this scale. The company uses native speaker translators and allowed for a second independent linguist review. This translation was compared with an informal translation of the document to ensure semantic equivalence.

Example of Use of Cross-Cultural Equivalence Framework to Establish Evidence of Scale Transferability

As per the COSMIN guideline for systematic reviews of patient reported outcome measures (*1*), cross-cultural validity was applied not only across nationalities or ethnicities, but also across outbreaks, and respondent profiles (i.e., those with and without lived experience of the disease).

A scale was considered to have been used with sufficient evidence of cross-cultural equivalence if the majority (i.e., at least three) of the categories defined by Stevelink and Van Brakel (*2*) (Appendix Table 1) met the criteria for 'extensive' and no categories met the criteria for 'minimal'. A scale was considered to have insufficient evidence of cross-cultural equivalence if it did not meet these criteria (Appendix Table 2).

References

1. Prinsen CAC, Mokkink LB, Bouter LM, Alonso J, Patrick DL, de Vet HCW, et al. COSMIN guideline for systematic reviews of patient-reported outcome measures. Qual Life Res. 2018;27:1147–57. PubMed https://doi.org/10.1007/s11136-018-1798-3

2. Stevelink SAM, van Brakel WH. The cross-cultural equivalence of participation instruments: a systematic review. Disabil Rehabil. 2013;35:1256–68. PubMed https://doi.org/10.3109/09638288.2012.731132

3. Jones N, Corrigan PW. Chapter 1: Understanding stigma. In: Corrigan PW, editor. The Stigma of disease and disability. Understanding causes and overcoming injustices. Washington (DC): American Psychological Association; 2014. p. 9–34.

4. Pescosolido BA, Martin JK. The Stigma Complex. Annu Rev Sociol. 2015;41:87–116. PubMed https://doi.org/10.1146/annurev-soc-071312-145702

5. Dar SA, Khurshid SQ, Wani ZA, Khanam A, Haq I, Shah NN, et al. Stigma in coronavirus disease-19 survivors in Kashmir, India: a cross-sectional exploratory study. PLoS One. 2020;15:e0240152. PubMed https://doi.org/10.1371/journal.pone.0240152

6. Kibria MG, Islam T, Islam MT, Kabir R, Ahmed S, Sultana P. Stigma and its associated factors among patients with COVID-19 in Dhaka City: evidence from a cross-sectional investigation. PeerJ. 2022;10:e14092. PubMed https://doi.org/10.7717/peerj.14092

7. Nair S, Joshi A, Aggarwal S, Adhikari T, Mahajan N, Diwan V, et al. Development & validation of scales to assess stigma related to COVID-19 in India. Indian J Med Res. 2022;155:156–64. PubMed https://doi.org/10.4103/ijmr.ijmr_2455_21

8. Adhikari T, Aggarwal S, Nair S, Joshi A, Diwan V, Stephen A, et al. Factors associated with COVID-19 stigma during the onset of the global pandemic in India: a cross-sectional study. Front Public Health. 2022;10:992046. PubMed https://doi.org/10.3389/fpubh.2022.992046

9. Mistry SK, Ali ARMM, Yadav UN, Huda MN, Rahman MM, Saha M, et al. Stigma toward people with COVID-19 among Bangladeshi older adults. Front Public Health. 2022;10:982095. PubMed https://doi.org/10.3389/fpubh.2022.982095

10. Al-Zamel LA, Al-Thunayan SF, Al-Rasheed AA, Alkathiri MA, Alamri F, Alqahtani F, et al. Validation and cultural adaptation of Explanatory Model Interview Catalogue (EMIC) in assessing stigma among recovered patients with COVID-19 in Saudi Arabia. Int J Environ Res Public Health. 2021;18:8261. PubMed https://doi.org/10.3390/ijerph18168261

11. Huang F, Chen WT, Shiu CS, Lin SH, Tun MS, Nwe TW, et al. Adaptation and validation of a culturally adapted HIV stigma scale in Myanmar. BMC Public Health. 2021;21:1663. PubMed https://doi.org/10.1186/s12889-021-11685-w

12. Li T, Bu H, Duan W. A brief measure of perceived courtesy and affiliate stigma on COVID-19: a study with a sample from China. Pers Individ Dif. 2021;180:110993. PubMed https://doi.org/10.1016/j.paid.2021.110993

13. Mlouki I, Zammit N, Ghammem R, Ben Fredj S, Bannour R, El Echi A, et al. Validity and reliability of a modified short version of a stigma scale for use among Tunisian COVID-19 patients after quarantine: a cross-sectional study. Health Sci Rep. 2022;5:e520. PubMed https://doi.org/10.1002/hsr2.520

14. Haddad C, Bou Malhab S, Malaeb D, Sacre H, Saadeh D, Mourtada V, et al. Stigma toward people with COVID-19 among the Lebanese population: a cross-sectional study of correlates and mediating effects. BMC Psychol. 2021;9:164. PubMed https://doi.org/10.1186/s40359-021-00646-y

15. Haddad C, Sacre H, Bou Malhab S, Malaeb D, Saadeh D, Abou Tayeh C, et al. A cross-sectional study of COVID-19-related bullying in a sample of Lebanese adults: scale validation, correlates, and mediating effect of fear and anxiety. BMC Psychol. 2021;9:137. PubMed https://doi.org/10.1186/s40359-021-00643-1

16. Bonetto C, Pace D, Bodini L, Colombi M, Van Bortel T, Lasalvia A. Development and psychometric validation of new questionnaires assessing experienced discrimination and internalised stigma among people with Covid-19. Epidemiol Psychiatr Sci. 2022;31:e37. PubMed https://doi.org/10.1017/S204579602200021X

17. Choi J, Kim KH. The differential consequences of fear, anger, and depression in response to COVID-19 in South Korea. Int J Environ Res Public Health. 2022;19:6723. PubMed https://doi.org/10.3390/ijerph19116723

18. Faghankhani M, Nourinia H, Rafiei-Rad AA, Adeli AM, Yeganeh MRJ, Sharifi H, et al. COVID-19 related stigma among the general population in Iran. BMC Public Health. 2022;22:1681. PubMed https://doi.org/10.1186/s12889-022-14039-2

19. Soleimani F, Aligholipour M, Aghal M, Aliafsari Mamaghani E. COVID-19 related perceived discrimination in medical settings, March and April 2020. Inquiry. 2021;58:469580211020884. PubMed https://doi.org/10.1177/00469580211020884

20. Akour A, AlMuhaissen SA, Nusair MB, Al-Tammemi AB, Mahmoud NN, Jalouqa S, et al. The untold story of the COVID-19 pandemic: perceptions and views towards social stigma and bullying in the shadow of COVID-19 illness in Jordan. SN Soc Sci. 2021;1:240. PubMed https://doi.org/10.1007/s43545-021-00252-0

21. Hossain MB, Alam MZ, Islam MS, Sultan S, Faysal MM, Rima S, et al. COVID-19 public stigma in the context of government-based structural stigma: A cross-sectional online survey of adults in Bangladesh. Stigma Health. 2021;6:123–33. https://doi.org/10.1037/sah0000305

22. Alatrany SSJ. COVID-19 related stigma, examining the view of the general public of stigma toward people with COVID-19 in Iraq. Int J Psychosoc Rehabil. 2020;24:7108–15. https://doi.org/10.37200/IJPR/V24I5/PR2020720

23. Alchawa M, Naja S, Ali K, Kehyayan V, Haddad PM, Bougmiza I. COVID-19 perceived stigma among survivors: a cross-sectional study of prevalence and predictors. Eur J Psychiatry. 2023;37:24–35. PubMed https://doi.org/10.1016/j.ejpsy.2022.08.004

24. Almoayad F, Mahboub S, Amer LB, Alrabiah A, Alhashem A. Stigmatisation of COVID-19 in Riyadh, Saudi Arabia: a cross-sectional study. Sultan Qaboos Univ Med J. 2021;21:525–31. PubMed https://doi.org/10.18295/squmj.4.2021.044

25. Babatunde OA, Owoicho SA, Sunday ST, Akande A, Yesufu BM, Akanbi IM, et al. An assessment of perceived stigmatization of patients infected with COVID-19 in the nation's epicenter of the pandemic: a cross-sectional study of residents of Agege, Lagos, Nigeria. West Afr J Med. 2021;38:1206–15. PubMed

26. Jiang T, Zhou X, Lin L, Pan Y, Zhong Y, Wang X, et al. COVID-19–related stigma and its influencing factors: a nationwide cross-sectional study during the early stage of the pandemic in China. BMJ Open. 2021;11:e048983. PubMed https://doi.org/10.1136/bmjopen-2021-048983

27. Nochaiwong S, Ruengorn C, Awiphan R, Kanjanarat P, Ruanta Y, Phosuya C, et al. COVID-19 Public Stigma Scale (COVID-PSS): development, validation, psychometric analysis and interpretation. BMJ Open. 2021;11:e048241. PubMed https://doi.org/10.1136/bmjopen-2020-048241

28. Osei E, Amu H, Appiah PK, Amponsah SB, Danso E, Oppong S, et al. Stigma and discrimination tendencies towards COVID-19 survivors: evidence from a nationwide population-based survey in Ghana. PLOS Glob Public Health. 2022;2:e0000307. PubMed https://doi.org/10.1371/journal.pgph.0000307

29. Preusting LC, Raadsen MP, Abourashed A, Voeten HACM, Wagener MN, de Wit E, et al. COVID-19 related stigma and health-protective behaviours among adolescents in the Netherlands: an explorative study. PLoS One. 2021;16:e0253342. PubMed https://doi.org/10.1371/journal.pone.0253342

30. Wilandika A, Gartika N, Salami S. Social stigma against individuals with COVID-19: scale development and validation. Health Psychol Behav Med. 2022;11:2155166. PubMed https://doi.org/10.1080/21642850.2022.2155166

31. El Rakhawy MY, Sabry NA, Elkhoreiby IA, Abdel Tawab AM, Mineo R, Hofstede A, et al. Do pandemics still cause mental health problems and social stigma? The case of COVID-19 in Egypt. Pak J Med Health Sci. 2021;15:904–9. https://pjmhsonline.com/2021/feb/904.pdf

32. Pantelic M, Ziauddeen N, Boyes M, O'Hara ME, Hastie C, Alwan NA. Long Covid stigma: estimating burden and validating scale in a UK-based sample. PLoS One. 2022;17:e0277317. PubMed https://doi.org/10.1371/journal.pone.0277317

33. Overholt L, Wohl DA, Fischer WA II, Westreich D, Tozay S, Reeves E, et al. Stigma and Ebola survivorship in Liberia: Results from a longitudinal cohort study. PLoS One. 2018;13:e0206595. PubMed https://doi.org/10.1371/journal.pone.0206595

34. Kelly JD, Weiser SD, Wilson B, Cooper JB, Glayweon M, Sneller MC, et al. Ebola virus disease-related stigma among survivors declined in Liberia over an 18-month, post-outbreak period: an observational cohort study. PLoS Negl Trop Dis. 2019;13:e0007185. PubMed https://doi.org/10.1371/journal.pntd.0007185

35. Kelly JD, Hoff NA, Spencer D, Musene K, Bramble MS, McIlwain D, et al. Neurological, cognitive, and psychological findings among survivors of Ebola virus disease from the 1995 Ebola outbreak in Kikwit, Democratic Republic of Congo: a cross-sectional study. Clin Infect Dis. 2019;68:1388–93. PubMed https://doi.org/10.1093/cid/ciy677

36. James PB, Wardle J, Steel A, Adams J. An assessment of Ebola-related stigma and its association with informal healthcare utilisation among Ebola survivors in Sierra Leone: a cross-sectional study. BMC Public Health. 2020;20:182. PubMed https://doi.org/10.1186/s12889-020-8279-7

37. Lieberman Lawry L, Stroupe Kannappan N, Canteli C, Clemmer W. Cross-sectional study of mental health and sexual behaviours for Ebola Survivors in Beni, Butembo and Katwa health zones of the Democratic Republic of Congo. BMJ Open. 2022;12:e052306. PubMed https://doi.org/10.1136/bmjopen-2021-052306

38. Antonaccio CM, Pham P, Vinck P, Collet K, Brennan RT, Betancourt TS. Fear, distress, and perceived risk shape stigma toward Ebola survivors: a prospective longitudinal study. BMC Public Health. 2021;21:2066. PubMed https://doi.org/10.1186/s12889-021-12146-0

39. Davidson MC, Lu S, Barrie MB, Freeman A, Mbayoh M, Kamara M, et al. A post-outbreak assessment of exposure proximity and Ebola virus disease-related stigma among community members in Kono District, Sierra Leone: A cross-sectional study. SSM Ment Health. 2022;2: 100064. PubMed https://doi.org/10.1016/j.ssmmh.2022.100064

40. Lee S, Chan LY, Chau AM, Kwok KP, Kleinman A. The experience of SARS-related stigma at Amoy Gardens. Soc Sci Med. 2005;61:2038–46. PubMed https://doi.org/10.1016/j.socscimed.2005.04.010

41. Gregorio ER Jr, Medina JRC, Lomboy MFTC, Talaga ADP, Hernandez PMR, Kodama M, et al. Knowledge, attitudes, and practices of public secondary school teachers on Zika virus disease: a basis for the development of evidence-based Zika educational materials for schools in the Philippines. PLoS One. 2019;14:e0214515. PubMed https://doi.org/10.1371/journal.pone.0214515

42. Usifoh SF, Odigie AE, Ighedosa SU, Uwagie-Ero EA, Aighewi IT. Lassa fever-associated stigmatization among staff and students of the University of Benin, Nigeria. J Epidemiol Glob Health. 2019;9:107–15. PubMed https://doi.org/10.2991/jegh.k.190514.001

43. Cénat JM, Noorishad PG, Dalexis RD, Rousseau C, Derivois D, Kokou-Kpolou CK, et al. Prevalence and risk factors of depression symptoms among rural and urban populations affected by Ebola virus disease in the Democratic Republic of the Congo: a representative cross-sectional study. BMJ Open. 2022;12:e053375. PubMed https://doi.org/10.1136/bmjopen-2021-053375

44. Cénat JM, Noorishad PG, Kokou-Kpolou CK, Dalexis RD, Hajizadeh S, Guerrier M, et al. Prevalence and correlates of depression during the COVID-19 pandemic and the major role of stigmatization in low- and middle-income countries: a multinational cross-sectional study. Psychiatry Res. 2021;297:113714. PubMed https://doi.org/10.1016/j.psychres.2021.113714

45. Cénat JM, Rousseau C, Bukaka J, Dalexis RD, Guerrier M. Severe Anxiety and PTSD symptoms among Ebola virus disease survivors and healthcare workers in the context of the COVID-19 pandemic in Eastern DR Congo. Front Psychiatry. 2022;13:767656. PubMed https://doi.org/10.3389/fpsyt.2022.767656

46. Mokkink LB, de Vet HCW, Prinsen CAC, Patrick DL, Alonso J, Bouter LM, et al. COSMIN risk of bias checklist for systematic reviews of patient-reported outcome measures. Qual Life Res. 2018;27:1171–9. PubMed https://doi.org/10.1007/s11136-017-1765-4

Appendix Table 1. Categories of cross-cultural equivalence used in an assessment of transferability of scales*

Equivalence	Definition
Conceptual	Achieved when the questionnaire has the same relationship to the underlying concept in both cultures, primarily in terms of domains included and the emphasis placed on different domains.
Item	Item equivalence exists when items estimate the same parameters on the latent trait being measured and when they are equally relevant and acceptable in both cultures.
Semantic	The transfer of meaning across languages, achieving a similar effect on respondents who speak different languages.
Operational	The possibility of using a similar questionnaire format, instructions, mode of administration and measurement methods.
Measurement	The psychometric properties of the adapted version of the participation measures are equivalent.

*Based on categories of equivalence from S.A.M. Stevelink and W.H. van Brakel's cross-cultural equivalence framework (*2*). All categories are rated minimal, partial, or extensive for each scale based on the evidence available from the relevant studies.

Appendix Table 2. Example of method to determine cross-cultural equivalence

Scale	Conceptual	Item	Semantic	Operational	Measurement	Total evidence
COVID-19-related Stigma Survey (cross-national use: India and Bangladesh)	Minimal	Partial	Minimal	Partial	Partial	Insufficient

Appendix Table 3. A Priori Conceptual Framework of Stigma*

Experiential target variants	Action-oriented target variants			
	Public stigma	Self stigma	Structural stigma	Label avoidance
Prejudice	Public prejudice	Self prejudice	Structural prejudice	Label avoidance due to prejudice
Discrimination	Public discrimination	Self discrimination	Structural discrimination	Label avoidance due to discrimination

*Diagrammatic representation of Jones and Corrigan (*3*) stigma typology; labels adopted from Pescosolido and Martin (*4*).

Appendix Table 4. Further details of included studies*

Scale	Study information			
	First author, y (reference no.)	Type of study in relation to scale‡ (Origin of items)	Target population; no.	Mode of administration (method)
COVID-19 stigma scales				
COVID-19-related Stigma Survey	S.A. Dar, 2020 (*5*)	Development (adapted from Ebola-related Stigma Scale)	Recovered patients; 91	Self-administered or interviewer-administered for illiterate participants (in person)
	G. Kibria, 2022 (*6*)	Validation	Recovered patients; 384	Interviewer-administered (in person or phone call)
COVID-19 Stigma Scale	S. Nair, 2022 (*7*)	Development (Adapted from HIV stigma scale, HIV stigma framework and news reports)	Recovered patients; 99	Interviewer-administered (phone call)
	T. Adhikari, 2022 (*8*)	Use	Recovered patients; 2,279	Interviewer-administered (phone call)
Community COVID-19 Stigma Scale	S. Nair, 2022 (*7*)	Development (Adapted from HIV stigma scale, HIV	Non-infected community members; 61	Interviewer-administered (phone call)

Scale	Study information			
	First author, y (reference no.)	Type of study in relation to scale‡ (Origin of items)	Target population; no.	Mode of administration (method)
		stigma framework and news reports)		
	T. Adhikari, 2022 (*8*)	Use	Non-infected community members; 2,279	Interviewer-administered (phone call)
Eight-item Stigma Scale	S.K. Mistry, 2022 (*9*)	Validation (Based in HIV scale adapted but not previously content validated)	All older adult community members; 1,045	Interviewer-administered (phone call)
Arabic Explanatory Model Interview Catalogue (EMIC)	L.A. Al-Zamel, 2021 (*10*)	Validation (Scale developed in context of leprosy and mental health stigma)	Recovered patients; 174	Interviewer-administered (phone call)
COVID-19 Stigma Instrument-Patients (CSI-P2)	F. Huang, 2021 (*11*)	Development (Adapted from HIV stigma scale)	Recovered patients; 151	Self-administered (online)
The Perceived Courtesy Stigma Sub-scale	T. Li, 2021 (*12*)	Development (Adapted from HIV stigma scales and other literature)	Non-infected community members; 2,812	Self-administered (online)
The Affiliate Stigma Sub-scale	T. Li, 2021 (*12*)	Development (Adapted from HIV stigma scales and other literature)	Non-infected community members; 2,812	Self-administered (online)
Modified 12-item HIV stigma scale	I. Mlouki, 2022 (*13*)	Development (Based on HIV stigma scale and literature review and qualitative interviews)	Current patients; 346	Interviewer-administered (phone call)
Stigma Discrimination Scale (SDS-11)	C. Haddad, 2021a (*14*)	Development (Adapted from HIV stigma scales)	All community members; 405	Self-administered (online)
Self-stigma Scale (SSS-15)	C. Haddad, 2021a (*14*)	Development (Adapted from HIV stigma scale and other literature)	Recovered patients; 49	Self-administered (online)
COVID-19 bullying scale	C. Haddad, 2021b (*15*)	Development (Adapted from existing bullying scales)	All community members; 405	Self-administered (online)
COVID-19 Experienced DISCrimination Scale (CEDISC)	C. Bonetto, 2022 (*16*)	Development (Adapted from HIV stigma scales)	Recovered patients; 579	Self-administered (online)
COvid-19 INternalised Stigma Scale (COINS)	C. Bonetto, 2022 (*16*)	Development (Adapted from HIV stigma scales)	Recovered patients; 519	Self-administered (online)
COVID-19 Responsibility Attribution scale	J.W. Choi, 2022 (*17*)	Development (Based on literature review, qualitative interviews, and author experience)	All community members; 1,000	Self-administered (online)
COVID-19 Attitudes scale	J.W. Choi, 2022 (*17*)	Development (Based on literature review, qualitative interviews, and author experience)	All community members; 1,000	Self-administered (online)
COVID-19-related enacted Stigma Questionnaire	M. Faghankhani, 2022 (*18*)	Development (Based on HIV and SARS stigma scales, social media analysis andqualitative interviews)	Non-infected community members; 630	Interviewer-administered (phone call)
Discrimination in Medical Settings Scale	F. Soleimani, 2021 (*19*)	Development (Source not specified)	Recovered patients; 176	N/S
30-item Bullying during the COVID-19 Pandemic Questionnaire	A. Akour, 2021 (*20*)	Development (Literature review and author experience)	Non-infected community members; 397	Self-administered (online)
Stigmatising Attitudes Scale	M.B. Hossain, 2021 (*21*)	Development (Source not specified)	All community members; 1,056	Self-administered (online)
COVID-19 Stigma Scale (COVID19SS)	S. Alatrany, 2020 (*22*)	Development (Literature review and author experience)	All community members; 953	Self-administered (online)
COVID-19 Perceived Stigma Scale-22 (CPSS-22)	M. Alchawa, 2022 (*23*)	Development (Adapted from HIV and cancer stigma scales)	Recovered patients; 404	Interviewer-administered (phone call)
Public Attitudes toward Stigma Questionnaire	F. Almoayad, 2020 (*24*)	Development (Based on literature review)	All community members; 847	Self-administered (online)

	Study information			
Scale	First author, y (reference no.)	Type of study in relation to scale‡ (Origin of items)	Target population; no.	Mode of administration (method)
Perceived Stigmatization of COVID-19 Scale	O.A. Babatunde, 2021 (*25*)	Development (Adapted from HIV stigma scale)	All community members; 333	Interviewer-administered (in person)
Public COVID-19-related Stigma toward Patients with COVID-19 Measure	T. Jiang, 2021 (*26*)	Development (Adapted from TB stigma scales)	All community members; 5,039	Self-administered (online)
Public COVID-19-related Stigma toward Wuhan People Measure	T. Jiang, 2021 (*26*)	Development (Adapted from TB stigma scales)	All community members; 4,628	Self-administered (online)
COVID-19 Public Stigma Scale	S. Nochaiwong, 2021 (*27*)	Development (Based on literature review and qualitative interviews)	All community members; 4,004	Self-administered (online)
Social stigma and discriminatory attitudes scale	E. Osei, 2022 (*28*)	Development (Based on HIV stigma literature)	All community members; 3,259	Interviewer-administered (in person)
Modified Measure of Disease-Related Stigma (MDRS) scale	L.C. Preusting, 2021 (*29*)	Development (Adapted from stigma scale used for HIV, cancer, and anorexia nervosa)	All adolescents in community; 380	Self-administered (online)
The social stigma scale	A. Wilandika, 2022 (*30*)	Development (Based on stigma theory)	Non-infected community members; 225	Self-administered (online)
COVID-19 related Social Stigma Scale	M.Y. El Rakhawy, 2021 (*31*)	Development (Based on focus groups and literature review)	All community members; 501	Self-administered (online)
Long COVID stigma scales				
Long COVID Stigma Scale (LCSS)	M. Pantelic, 2022 (*32*)	Development (Based on chronic illness stigma scales and qualitative interviews)	Current patients; 888	Self-administered (online)
EVD stigma scales				
Ebola-related Stigma Scale	L. Overholt, 2018 (*33*)	Development (Adapted from HIV stigma scales)	Recovered patients; 299	Interviewer-administered (in person)
7-item EVD-related stigma index	J.D. Kelly, 2019a (*34*)	Development (Adapted from HIV stigma scale)	Recovered patients; 859	Interviewer-administered (in person)
	J.D. Kelly, 2019b (*35*)	Use	Recovered patients and close contacts; 207	Interviewer-administered (in person)
Ebola-related stigma instrument	P.B. James, 2020 (*36*)	Development (Adapted from HIV stigma scale)	Recovered patients; 358	Self-administered or interviewer-administered for illiterate participants (in person)
EVD-related stigma scale	L.L. Lawry, 2022 (*37*)	Development (Based on unspecified existing instruments)	Recovered patients, partners and noninfected community members; 399	Interviewer-administered (in person)
Stigma toward EVD Survivors Scale	C.M. Antonaccio, 2021 (*38*)	Development (Adapted from HIV stigma scale)	All community members; 1,008	Interviewer-administered (in person)
EVD Stigma Index	M.C. Davidson, 2022 (*39*)	Development (Adapted from HIV stigma scale)	Non-infected community members; 538	Self-administered (in person)
SARS stigma scales				
SARS Social Life and Services Stigma Self-report Questionnaire	S. Lee, 2005 (*40*)	Development (Based on focus groups)	All community members; 903	Self-administered (posted)
SARS Discrimination in the Workplace Self-report Questionnaire	S. Lee, 2005 (*40*)	Development (Based on focus groups)	All community members; 903	Self-administered (posted)
Zika Virus Disease stigma scales				

Scale	Study information			
	First author, y (reference no.)	Type of study in relation to scale‡ (Origin of items)	Target population; no.	Mode of administration (method)
Modified Version of the Knowledge, Attitudes, and Practices Survey Tool on Zika Virus Disease	E.R. Gregorio, 2019 (*41*)	Development (Based on WHO survey tool)	All community-based teachers; 609	Self-administered (in person)
Lassa Fever stigma scales				
Lassa fever-associated stigmatization scale	S.F. Usifoh, 2019 (*42*)	Development (Source not specified)	All university students and staff in community; 600	Self-administered (in person)
Stigma scales used across multiple diseases				
Stigmatization related to EVD and COVID-19 scale	J.M. Cénat, 2022a (*43*)	Development, validation and use (Based on WHO reports and the Social science and behavioral data compilation)	All community members; 1,614 then 824	Interviewer-administered (in person)
	J.M. Cénat, 2021 (*44*)	Validation and use for COVID-19 (adapted in laboratory with a panel of experts according to WHO studies)	All community members; 1,267	Self-administered (online)
	J.M. Cénat, 2022b (*45*)	Validation and use	Recovered patients and healthcare workers; 563	Interviewer-administered (in person)

*When a name was not formally given to a scale, we used the most commonly used terms. Each set of items that could be combined to form a composite score were regarded as a scale. If a sub-scale could not be combined with other sub-scales to form a composite score it was regarded as a unique scale. All scale development included initial validation. EVD, Ebola virus disease; NS, not specified.

Appendix Table 5. Overview of included scales and respective studies*

Scale	First author, y (reference no.)	Country (Language(s) of administration)	Study methods in relation to scale†	Quality rating (COSMIN Risk of Bias Assessment)‡
COVID-19 stigma scales				
COVID-19-related Stigma Survey	S.A. Dar, 2020 (*5*)	India (NS)	Scale development	Inadequate
	G. Kibria, 2022 (*6*)	Bangladesh (Bengali)	Content validation	Doubtful
COVID-19 Stigma Scale	S. Nair, 2022 (*7*)	India (Hindi, Tamil, Marathi, Odia, and Assamese)	Scale development	Inadequate
			Test-retest reliability testing	Doubtful
	T. Adhikari, 2022 (*8*)	India (Hindi, Tamil, Marathi, Odia, and Assamese)	Scale use only	NA
Community COVID-19 Stigma Scale	S. Nair, 2022 (*7*)	India (Hindi, Tamil, Marathi, Odia, and Assamese)	Scale development	Inadequate
			Test-retest reliability testing	Adequate
	T. Adhikari, 2022 (*8*)	India (Hindi, Tamil, Marathi, Odia, and Assamese)	Scale use only	NA
Eight-item Stigma Scale	S.K. Mistry, 2022 (*9*)	Bangladesh (Bengali)	Content validation	Inadequate
Arabic Explanatory Model Interview Catalogue (EMIC)	L.A. Al-Zamel, 2021 (*10*)	Saudi Arabia (Arabic)	Content validation	Doubtful
			Structural validity testing	Adequate
			Internal consistency testing	Adequate
COVID-19 Stigma Instrument-Patients (CSI-P2)	F. Huang, 2021 (*11*)	China (Chinese)	Scale development	Inadequate
			Structural validity testing	Adequate

Scale	First author, y (reference no.)	Country (Language(s) of administration)	Study methods in relation to scale†	Quality rating (COSMIN Risk of Bias Assessment)‡
			Internal consistency testing	Adequate
The Perceived Courtesy Stigma Sub-scale	T. Li, 2021 (*12*)	China (Chinese)	Scale development	Inadequate
			Structural validity testing	Very good
			Internal consistency testing	Very good
			Test-retest reliability testing	Adequate
			Hypotheses testing	Adequate
The Affiliate Stigma Sub-scale	T. Li, 2021 (*12*)	China (Chinese)	Scale development	Inadequate
			Structural validity testing	Very good
			Internal consistency testing	Very good
			Test-retest reliability testing	Adequate
			Hypotheses testing	Adequate
Modified 12-item HIV stigma scale	I. Mlouki, 2022 (*13*)	Tunisia (Tunisian Arabic)	Scale development	Doubtful
			Structural validity testing	Very good
			Internal consistency testing	Very good
Stigma Discrimination Scale (SDS-11)	C. Haddad, 2021a (*14*)	Lebanon (English and Arabic)	Scale development	Inadequate
			Structural validity testing	Very good
			Internal consistency testing	Very good
Self-stigma Scale (SSS-15)	C. Haddad, 2021a (*14*)	Lebanon (English and Arabic)	Scale development	Inadequate
			Structural validity testing	Inadequate
COVID-19 bullying scale	C. Haddad, 2021b (*15*)	Lebanon (Arabic)	Scale development	Inadequate
			Structural validity testing	Very good
			Internal consistency testing	Very good
COVID-19 Experienced DISCrimination Scale (CEDISC)	C. Bonetto, 2022 (*16*)	Italy (Italian)	Scale development	Inadequate
			Structural validity testing	Very good
			Internal consistency testing	Very good
			Test-retest reliability testing	Adequate
COvid-19 INternalised Stigma Scale (COINS)	C. Bonetto, 2022 (*16*)	Italy (Italian)	Scale development	Inadequate
			Structural validity testing	Very good
			Internal consistency testing	Very good
			Test-retest reliability testing	Adequate
COVID-19 Responsibility Attribution scale	J.W. Choi, 2022 (*17*)	South Korea (NS)	Scale development	Inadequate
COVID-19 Attitudes scale	J.W. Choi, 2022 (*17*)	South Korea (NS)	Scale development	Inadequate
COVID-19-related enacted Stigma Questionnaire	M. Faghankhani, 2022 (*18*)	Iran (Persian)	Scale development	Doubtful
			Structural validity testing	Adequate

Scale	First author, y (reference no.)	Country (Language(s) of administration)	Study methods in relation to scale†	Quality rating (COSMIN Risk of Bias Assessment)‡
			Internal consistency testing	Adequate
Discrimination in Medical Settings Scale	F. Soleimani, 2021 (*19*)	Iran (NS)	Scale development	Inadequate
30-item Bullying during the COVID-19 Pandemic Questionnaire	A. Akour, 2021 (*20*)	Jordan (Arabic)	Scale development	Inadequate
Stigmatising Attitudes Scale	M.B. Hossain, 2021 (*21*)	Bangladesh (Bengali)	Scale development	Inadequate
COVID-19 Stigma Scale (COVID19SS)	S. Alatrany, 2020 (*22*)	Iraq (Arabic)	Scale development	Inadequate
			Structural validity testing	Inadequate
COVID-19 Perceived Stigma Scale-22 (CPSS-22)	M. Alchawa, 2022 (*23*)	Qatar (Arabic and English)	Scale development	Inadequate
Public Attitudes toward Stigma Questionnaire	Almoayad, F (2020)	Saudi Arabia (Arabic)	Scale development	Inadequate
			Structural validity testing	Inadequate
Perceived Stigmatization of COVID-19 Scale	O.A. Babatunde, 2021 (*25*)	Nigeria (NS)	Scale development	Inadequate
Public COVID-19-related Stigma toward Patients Measure	T. Jiang, 2021 (*26*)	China (Mandarin)	Scale development	Inadequate
Public COVID-19-related Stigma toward Wuhan People Measure	T. Jiang, 2021 (*26*)	China (Mandarin)	Scale development	Inadequate
COVID-19 Public Stigma Scale	S. Nochaiwong, 2021 (*27*)	Thailand (Thai)	Scale development	Doubtful
			Structural validity testing	Very good
			Internal consistency testing	Very good
			Hypotheses testing	Very good
			Test-retest reliability testing	Adequate
Social stigma and discriminatory attitudes scale	E. Osei, 2022 (*28*)	Ghana (Local languages)	Scale development	Inadequate
Modified Measure of Disease-Related Stigma (MDRS) scale	L.C. Preusting, 2021 (*29*)	Netherlands (Dutch)	Scale development	Inadequate
			Structural validity testing	Very good
			internal consistency testing	Very good
The social stigma scale	A. Wilandika, 2022 (*30*)	Indonesia (NS)	Scale development	Inadequate
			Structural validity testing	Very good
			Internal consistency testing	Very good
COVID-19 related Social Stigma Scale	M.Y. El Rakhawy, 2021 (*31*)	Egypt (Arabic)	Scale development	Inadequate
			Structural validity testing	Inadequate
Long COVID stigma scales				
Long COVID Stigma Scale (LCSS)	M. Pantelic, 2022 (*32*)	United Kingdom (NS)	Scale development	Inadequate
			Structural validity testing	Very good
			Internal consistency testing	Very good
			Hypotheses testing	Adequate
EVD stigma scales				
Ebola-related Stigma Scale	L. Overholt, 2018 (*33*)	Liberia (Liberian English)	Scale development	Inadequate

Scale	First author, y (reference no.)	Country (Language(s) of administration)	Study methods in relation to scale†	Quality rating (COSMIN Risk of Bias Assessment)‡
7-item EVD-related stigma index	J.D. Kelly, 2019a (*34*)	Liberia (Local languages)	Scale development	Inadequate
			Structural validity testing	Very good
			Internal consistency testing	Very good
	J.D. Kelly, 2019b (*35*)	DRC (NS)	Internal consistency testing	Very good
Ebola-related stigma instrument	P.B. James, 2020 (*36*)	Sierra Leone (NS)	Scale development	Inadequate
EVD-related stigma scale	L.L. Lawry, 2022 (*37*)	Democratic Republic of the Congo (Kiswahili and Kinande)	Scale development	Inadequate
Stigma toward EVD Survivors Scale	C.M. Antonaccio, 2021 (*38*)	Sierra Leone (Krio)	Scale development	Inadequate
EVD Stigma Index	M.C. Davidson, 2022 (*39*)	Sierra Leone (N/S)	Scale development	Inadequate
			Structural validity testing	Inadequate
SARS stigma scales				
SARS Social Life and Services Stigma Self-report Questionnaire	S. Lee, 2005 (*40*)	Hong Kong (Chinese)	Scale development	Doubtful
SARS Discrimination in the Workplace Self-report Questionnaire	S. Lee, 2005 (*40*)	Hong Kong (Chinese)	Scale development	Doubtful
Zika Virus Disease stigma scales				
Modified Version of the Knowledge, Attitudes, and Practices Survey Tool on Zika Virus Disease	E.R. Gregorio, 2019 (*41*)	Philippines (Filipino)	Scale development	Inadequate
Lassa fever stigma scales				
Lassa fever-associated stigmatization scale	S.F. Usifoh, 2019 (*42*)	Nigeria (NS)	Scale development	Inadequate
Stigma scales used across multiple diseases				
Stigmatization related to EVD and COVID-19 scale	J.M. Cénat, 2022a (*43*)	DRC (Lingala)	Scale development	Doubtful
	J.M. Cénat, 2021 (*44*)	DRC, Haiti, Rwanda, and Togo (French, Creole, English, and Kinyarwanda)	Content validation	Inadequate
	J.M. Cénat, 2022b (*45*)	DRC (Swahili, Lingala, Tshiluba, Kikongo, French, and English)	Content validation	Doubtful

*When a name was not formally given to a scale, we used terms most commonly used to refer to the scale. Each set of items that could be combined to form a composite score were regarded as a scale. If a subscale could not be combined with other subscales to form a composite score, it was regarded as a unique scale. DRC, Democratic Republic of the Congo; EVD, Ebola virus disease; NA, not applicable; NS, not specified.
†All scale development included initial validation. All studies included use of the scale.
‡The COSMIN Risk of Bias Checklist uses a modular approach of reviewing studies in relation to scale development and validation (*46*).

Appendix Table 6. Overview of psychometric properties of included scales

Scales	Overall rating of psychometric properties according to COSMIN criteria for good measurement properties: Content validity	Structural validity	Internal consistency	Test-retest Reliability	Hypotheses testing	Quality of evidence (COSMIN modified GRADE approach)
The Perceived Courtesy Stigma Sub-scale	?	+	+	+	+	Very low (CV); high (SV, IC); moderate (HT, RTR)
The Affiliate Stigma Sub-scale	?	+	+	+	+	Very low (CV); high (SV, IC); moderate (HT, RTR)
COVID-19 Public Stigma Scale	?	+	–	+	+	Low (CV); high (SV, IC, HT); moderate (TRT)
COVID-19 Stigma Instrument-Patients (CSI-P2)	?	+	+		+	Very low (CV); moderate (SV, IC); high (HT)
Long COVID Stigma Scale (LCSS)	?	+	–		+	Very low (CV); high (SC, IC); moderate (HT)
COvid-19 INternalised Stigma Scale (COINS)	?	+	+	–	ND	Very low (CV); high (SV, IC); moderate (RTR)
COVID-19 Experienced DISCrimination Scale (CEDISC)	?	+	+	–	ND	Very low (CV); high (SV, IC); moderate (RTR)
7-item EVD-related stigma index	?	?	?	ND	ND	Very low (CV); high (SC, IC)
Arabic Explanatory Model Interview Catalogue (EMIC)	?	+	+	ND	ND	Low (CV); high (SC, IC)
Modified 12-item HIV stigma scale	±	+	+	ND	ND	Very low (CV); high (SV, IC)
Stigma Discrimination Scale (SDS-11)	?	+	+	ND	ND	Very low (CV); high (SV, IC)
Self-stigma Scale (SSS-15)	?	?	?	ND	ND	Very low (CV, SV)
COVID-19 bullying scale	?	+	+	ND	ND	Very low (CV); high (SV, IC)
EVD Stigma Index	?	?	?	ND	ND	Very low (CV, SV)
COVID-19-related enacted Stigma Questionnaire	±	–	?	ND	ND	Low (CV); moderate (SV, IC)
COVID-19 Stigma Scale (COVID19SS)	?	?	?	ND	ND	Very low (CV, SV)
Public Attitudes toward Stigma Questionnaire	?	?	?	ND	ND	Very low (CV, SV)
Modified Measure of Disease-Related Stigma (MDRS) scale	?	+	–	ND	ND	Very low (CV); high (SV, IC)
COVID-19 related Social Stigma Scale	?	?	?	ND	ND	Very low (CV, SV, IC)
COVID-19 Stigma Scale	?	ND	ND	–	ND	Very low (CV); low (TRR)
Community COVID-19 Stigma Scale	?	ND	ND	–	ND	Very low (CV); moderate (RTR)
Stigmatization related to EVD and COVID-19 scale	±	ND	ND	ND	ND	Moderate
Eight-item Stigma Scale	?	ND	ND	ND	ND	Very low
Ebola-related stigma instrument	?	ND	ND	ND	ND	Very low
COVID-19 Responsibility Attribution scale	?	ND	ND	ND	ND	Very low
COVID-19 Attitudes scale	?	ND	ND	ND	ND	Very low
SARS Social Life and Services Stigma Self-report Questionnaire	?	ND	ND	ND	ND	Low
SARS Discrimination in the Workplace Self-report Questionnaire	?	ND	ND	ND	ND	Low
Stigma toward EVD Survivors Scale	?	ND	ND	ND	ND	Very low
Discrimination in Medical Settings Scale	?	ND	ND	ND	ND	Very low (CV)
30-item Bullying during the COVID-19 Pandemic Questionnaire	?	ND	ND	ND	ND	Very low (CV)
Stigmatising Attitudes Scale	?	ND	ND	ND	ND	Very low (CV)

Scales	Overall rating of psychometric properties according to COSMIN criteria for good measurement properties					Quality of evidence (COSMIN modified GRADE approach)
	Content validity	Structural validity	Internal consistency	Test-retest Reliability	Hypotheses testing	
COVID-19 Perceived Stigma Scale-22 (CPSS-22)	?	ND	ND	ND	ND	Very low
Perceived Stigmatization of COVID-19 Scale	?	ND	ND	ND	ND	Very low
Modified Version of the Knowledge, Attitudes, and Practices Survey Tool on Zika Virus Disease	?	ND	ND	ND	ND	Very low
Public COVID-19-related Stigma toward Patients with COVID-19 Measure	?	ND	ND	ND	ND	Very low
Public COVID-19-related Stigma toward Wuhan People Measure	?	ND	ND	ND	ND	Very low
EVD-related stigma scale	?	ND	ND	ND	ND	Very low
Social stigma and discriminatory attitudes scale	?	ND	ND	ND	ND	Very low
Lassa fever-associated stigmatization scale	?	ND	ND	ND	ND	Very low
The social stigma scale	?	ND	ND	ND	ND	Very low (CV); high (SV, IC)
COVID-19-related Stigma Survey	?	ND	ND	ND	ND	Low
Ebola-related Stigma Scale	?	ND	ND	ND	ND	Very low

*Adapted from COSMIN Guidelines for Systematic Reviews Appendix Table 3 (*1*). No scales assessed measurement error or responsiveness. Measurement invariance assessed as part of cross-cultural validity in Appendix Table 1. CV, content validity; HT, hypotheses testing (external construct validity); IC, internal consistency; ND, no data; SV, structural validity; TRT, test-retest reliability; +, sufficient; –, insufficient; ±, inconsistent; ?, indeterminate.

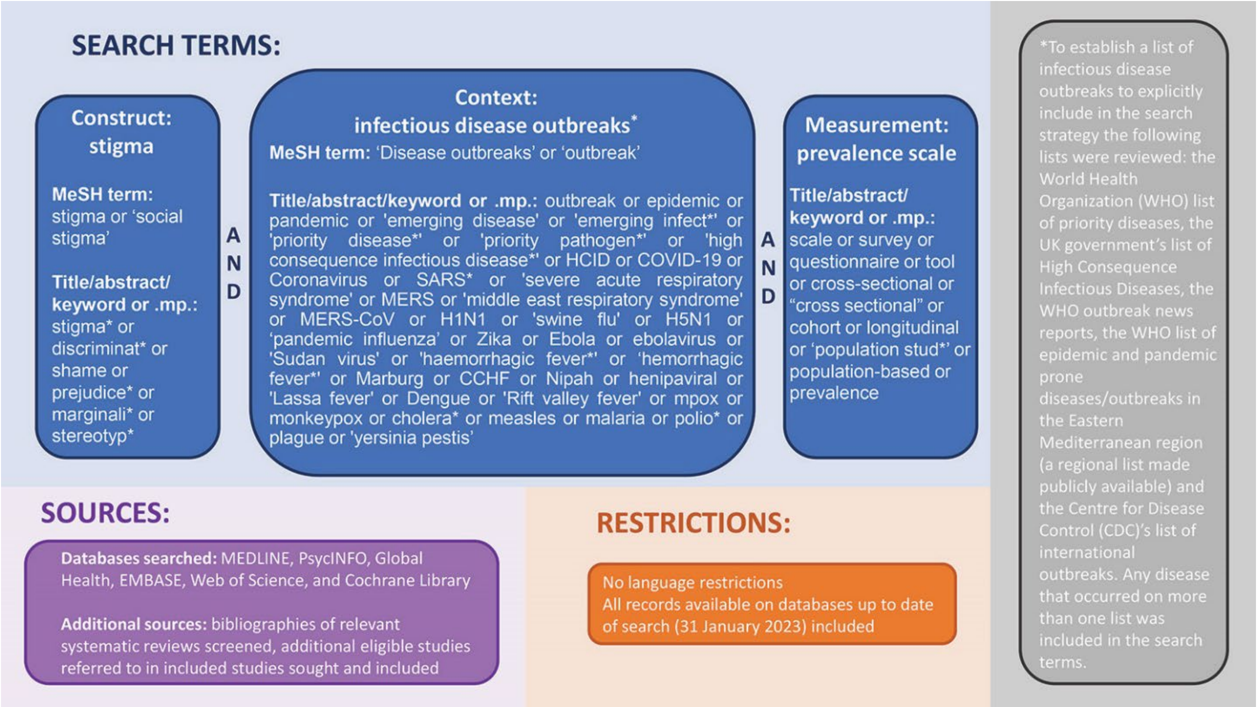

Appendix Figure. Search strategy used in a systematic review of scales for measuring infectious disease–related stigma.
